# Supplementary material for: Support needs and adaptive behavior surveys: Services prediction and relationship
Source: PLoS One. 2025 Sep 19;20(9):e0294539. doi: 10.1371/journal.pone.0294539 (PMC12448967; doi:10.1371/journal.pone.0294539)
Supplement: S1 File — Supplement 1. This document details the factors used to determine IBAs and includes a description of how the state decides the IBA budget based on living situation and age. (DOCX) [file pone.0294539.s001.docx]

**Supplement 1**

The state uses three factors to determine IBAs*:* 1. An assessed Level of Service (LOS) score assigned to a participant based on their ICAP scores; 2. The participant’s living situation: family home, independently or semi-independently, or in community living services; and 3. The participant’s age: over 21 or under 21 and in school. For #1, there are two formulas that are used to determine the level of service score involving parts of the ICAP:

Level of Service = (−0.0619 ×ICAP Service Score) + 6.827

Level of Service = (−0.2232 × General Score) + (−4.21 × 10−8 ×Personal Living3) + (−8.12 ×10−10 ×Personal Living3 × General Score) + 7.2457

The higher of the two LOS scores from these formulas is used in combination with living situation and age to estimate number of Medicaid units (see Supplement Table 1).

**Supplement Table 1**

*State decided IBA budget based on living situation and age.*


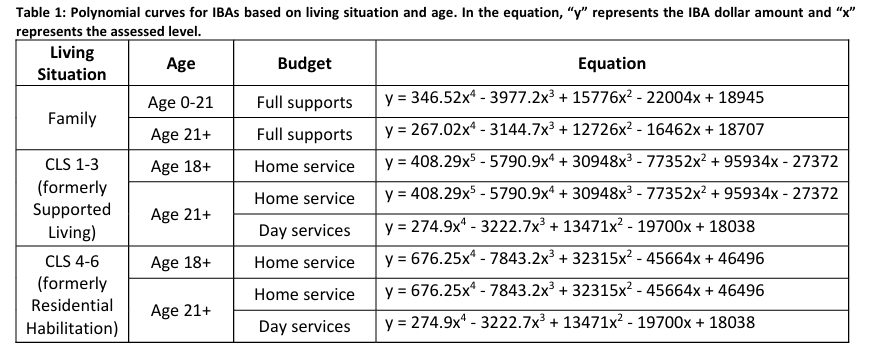


Note: This table is from the state’s algorithm for determining IBA ([Blinded]). X defines the assessed Level of Service (LOS) based parts of the ICAP.
